# The early influence of COVID-19 pandemic-associated restrictions on pain, mood, and everyday life of patients with painful polyneuropathy

**DOI:** 10.1097/PR9.0000000000000858

**Published:** 2020-10-14

**Authors:** Dilara Kersebaum, Sophie-Charlotte Fabig, Manon Sendel, Juliane Sachau, Josephine Lassen, Stefanie Rehm, Philipp Hüllemann, Ralf Baron, Janne Gierthmühlen

**Affiliations:** Division of Neurological Pain Research and Therapy, Department of Neurology, University Hospital of Schleswig-Holstein, Campus Kiel, Germany

**Keywords:** COVID-19, Neuropathic pain, Questionnaires, Chronic pain, Social isolation, Psychological

## Abstract

**Introduction:**

The SARS-Cov-2 pandemic requires special attention on its psychological effects and the impact on patients with chronic pain.

**Objectives:**

This study aimed at examining the influence of the COVID-19 pandemic-associated regulations initiated by the German government on pain intensity and characteristics, emotional well-being, and everyday life of patients with painful polyneuropathy.

**Methods:**

Forty-three patients (well assessed with questionnaires before the pandemic and without change of their health status between baseline and current assessment) were investigated with validated, self-reported questionnaires and COVID-19-specific items 2 weeks after the regulations came into effect.

**Results:**

Pain intensity remained stable or even improved like the neuropathic pain symptom inventory total score (t0: 33.54 ± 20.48 vs t1: 27.38 ± 16.16, *P* = 0.008). Only 11.6% reported a pandemic-associated pain worsening. Rumination scores of the Pain Catastrophizing Scale were lower during t1 compared to before the pandemic regulations (t0: 7.81 ± 4.70, t1: 6.49 ± 4.39; *P* = 0.030). Interestingly, pain ratings for the last 7 days were higher in patients with a changed social life compared to those without (−1.63 ± 1.60 vs 0.31 ± 1.83; *P* = 0.01). Quality of life was decreased and helplessness increased in those with higher pain ratings.

**Conclusion:**

Results suggest a shift of attention from the chronic pain condition towards the imminent threat of a global pandemic. As the impacts of the pandemic are persistent and evolving, the development of the measured parameters in the forthcoming weeks will be of great interest.

## Abstract 2

Supplemental Digital Content is Available in the Text.

Validated questionnaires revealed a shift of attention from chronic pain towards the COVID-19 pandemic in patients with chronic painful polyneuropathy.

## 1. Introduction

On January 7, 2020, a strain of coronavirus (SARS-CoV-2) that had not been detected in humans before has been identified to be responsible for the occurrence of several recent cases of acute respiratory distress syndrome in Wuhan City (Hubei province, China). To date, the disease caused by SARS-CoV-2 (coronavirus disease 2019, COVID-19) has evolved into a pandemic. With 25,029,408 cases (according to the respective case definition and testing rate of each country) and at least 843,158 fatal outcomes by finalization of this article, the COVID-19 pandemic poses vast challenges for international healthcare systems.^15^

The World Health Organization (WHO) has published technical guidelines concerning COVID-19.^45^The regulations taken by each country to contain and manage the outbreak in the spring of 2020 were individual but had one thing in common: an extensive impact on private, professional, and public life, as well as education, economy, and patient care,^13^ leading to social distancing, existential fear through loss of income or unemployment, and a feeling of uncertainty^42^ with unforeseeable long-term effects.

Previous infectious disease outbreaks have been shown to be associated with increased rates of depression, anxiety, and generalised fear.^30,37^ A study examining the long-term psychiatric morbidities among SARS survivors found posttraumatic stress disorder and depressive disorders to be the most prevalent long-term psychiatric conditions.^30^ This is also expected for the current pandemic,^43^ and the WHO has published recommendations to support mental and psychosocial well-being in different target groups during the outbreak.^47^

Chronic pain, affecting up to 20% of the world's population,^23^ occurs in comorbidity with depression^33^ and concerns multiple fields, eg, social life and economy.^19^ The biopsychosocial approach of pain describes pain and disability as a multidimensional, dynamic integration among physiological, psychological, and social factors that reciprocally influence each other.^31^ It has been reported that pain interference is influenced by social isolation, and therapeutic interventions aimed at increasing social connection can potentially reduce the impact of pain on engagement with activities.^26^ Consequently, chronic pain patients might exceptionally be affected by the pandemic due to medical concerns and the change of social life. It was therefore our hypothesis that in a group of patients with painful polyneuropathy, the emotional well-being and consequently the experienced pain intensity would deteriorate due to the pandemic and social isolation. This study aimed at the examination of this hypothesis in the early phase of the pandemic in Germany (for exact regulations of the German government, see Appendix 1, available at http://links.lww.com/PR9/A84).

## 2. Methods

### 2.1. Study design

**Figure 1.**
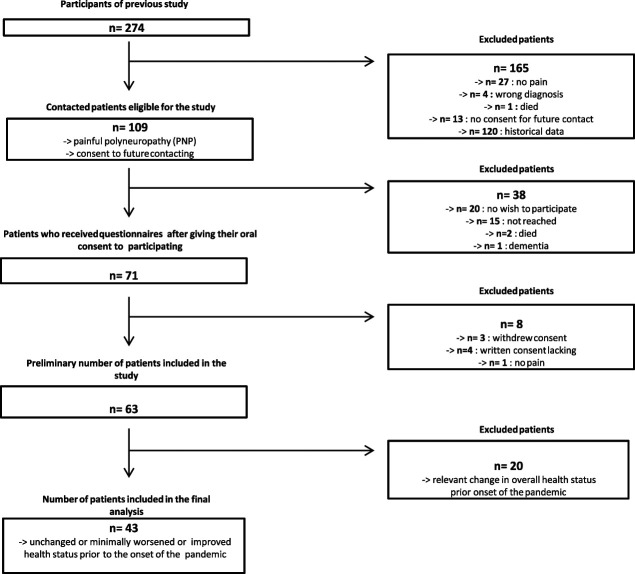
Recruitment of study cohort.

Patients with confirmed painful polyneuropathy who had been well-characterized clinically and with questionnaires for a former study were contacted by telephone and asked about possible participation in a new study (Fig. 1). In case of consent, the patient information and agreement were sent along with a set of standardized questionnaires relating to pain, emotional well-being, sleep, and physical activity as well as pandemic-associated questions about changes in daily life due to the pandemic. To account for possible spontaneous changes in the above-mentioned parameters on overall state of health in the time between first assessment in the context of the former study and the current study, patients were asked to rate their overall health status for the time point before the corona pandemic (the time until the end of February 2020) compared to the time point of assessment for the former study on a seven-staged Patient Global Impression of Change^20^ Likert-scale with 1 = very much improved, 2 = moderately improved, 3 = minimally improved, 4 = unchanged, 5 = minimally worse, 6 = moderately worse, and 7 = very much worse. The exact date of the patients' last visit was noted on the Patient Global Impression of Change questionnaire. For the analysis, only patients who reported an unchanged or minimally worsened or improved health status were included to exclude patients with a change of health status before the pandemic due to other reasons (Fig. 1).

All contacted patients had probable (presence of a combination of symptoms and signs of neuropathy including any 2 or more of the following: neuropathic symptoms, decreased distal sensation, or unequivocally decreased or absent ankle reflexes) or confirmed (presence of an abnormality of nerve conduction or validated measure of small fiber neuropathy with class 1 evidence with corresponding symptoms) neuropathy according to Tesfaye et al.^41^ and neuropathic pain was diagnosed according to the NeuPSIG algorithm.^16^ Only patients with probable or definite neuropathic pain according to the NeuPSIG algorithm were included in the analysis. Patients with any additional painful comorbidity that could influence questionnaire results were excluded from participation in the study.

The questionnaires were sent to the patients on April 3rd, ie, approximately 2 weeks after the governmental regulations became effective (eg, prohibition of private events of any kinds). By then, we expected potential measurable changes that might have occurred as a consequence of the regulations. Governmental regulations were stable during the time when the patients received and completed the questionnaires.

All patients provided written informed consent to participate in the study. The study was performed in accordance with the Declaration of Helsinki, approved by the local ethics committee (AZ D453/20) and registered at the German Clinical Trials Register (DRKS00021254), also accessible through the WHO Internationals Clinical Trials Registry Platform.

### 2.2. Questionnaires

Demographic data (ie, age, sex, average pain intensity, pain relief, comorbidities, pain medication, pain location, and cause of neuropathy) of the patients were collected.

### 2.3. Assessment of pain

#### 2.3.1. Pain intensity and characteristics

Pain presence, frequency, current analgesic medication, and pain relief were assessed with the Brief Pain Inventory (BPI).^9^ Originally developed for the measurement of cancer-related pain, the BPI is today validated in many languages and widely used for the assessment of chronic pain diseases^10^ and for acute pain conditions.^28^ Pain severity, impact on daily function (not assessed in this study), pain location, pain medications, and the amount of pain relief in the past 24 hours and the past week are assessed either self-reportedly or through an interview. Pain intensity was calculated with the BPI pain severity subscale ranging from 0 (no pain) to 10 (maximum intensity). The scale was then interpreted to mild (1–3), moderate (4–6), and severe pain (7–10). An interpretation of this numeric scale into mild, moderate, and severe is common. Because the BPI User Guide does not recommend specific cutoff points,^8^ we chose this categorization because the intensity “4” is usually considered to be the threshold for inclusion in pain studies.^3^

The Neuropathic Pain Symptom Inventory (NPSI), a self-administered questionnaire specifically developed to evaluate the severity of neuropathic pain, consists of 10 items plus 2 temporal items that are rated on a scale from 0 to 10 (0 = none, 10 = maximal imaginable intensity). Higher scores indicate more severe neuropathic pain.

#### 2.3.2. Pain interference

The Patient Reported Outcomes Measurement Information System (PROMIS) Short Form v1.0—Pain Interference 6a^1^ is a self-reported, 6-item measure for the assessment of consequences of pain on life's relevant aspects. There are 5 response options to each item, ranging from 1 to 5 (1 = not at all, 5 = very much). After the raw score is calculated by summing the score of each question, it is converted into a T-Score through a conversion table, resulting in a standardised score with a mean of 50 and an SD of 10. Values >60 are considered abnormal. Higher scores indicate a higher symptom/disease burden.

### 2.4. Assessment of emotional well-being and sleep

Patient Reported Outcomes Measurement Information System questionnaires^7^ have been used to examine sleep disturbance (PROMIS Short Form v1.0 Sleep Disturbance 6a^6^), fatigue (PROMIS Short Form v1.0—Fatigue 4a^27^), anxiety (PROMIS Short Form v1.0 -Anxiety 6a^35^), and depression (PROMIS Short Form v1.0—Depression 6a^35^). Their scoring works similarly to the above-mentioned system for the pain interference measure.

### 2.5. Assessment of personality characteristics

#### 2.5.1. Pain catastrophizing

The Pain Catastrophizing Scale (PCS)^38^ was used to assess pain catastrophising traits. It consists of 13 statements describing different thoughts and feelings that may be associated with pain. These statements are to be rated on a 5-point Likert scale (ranging from 0 “not at all” to 4 “all the time”), a total score is obtained by scoring all the items. Higher scores indicate greater pain catastrophizing.

#### 2.5.2. Personality

The factor IV 10-item scale of the International Personality Item Pool's (IPIP)^17^ is a representation of the Goldberg^18^ markers for Emotional Stability. The patient is supposed to rate each item corresponding to the level of accordance with his own personality (ranging from very inaccurate to accurate). For the first 2 questions, 1 corresponds to “very inaccurate” and 5 to “very accurate,” whereas for the remaining, it is reverse. A sum score is calculated. For the interpretation of individual scores, the mean and SD for a sample of persons (same sex and particular age range) is calculated. Scores within one-half SD of the mean can be interpreted as “average”, and outside that range as “low” or “high”.^24^

### 2.6. Assessment of quality of life

Quality of life was measured with the EQ-5D-5L,^21,40^ one of the most frequently used instruments for the evaluation of health-related quality of life. Besides a visual analogue scale (EQ VAS) where the patient is asked to rate the overall health state on a 0 to 100 scale, there is a descriptive system containing 5 different dimensions (mobility, self-care, usual activities, pain/discomfort, and anxiety/depression) that are to be rated on a five-point Likert scale (ranging from 1 to 5). Patients are asked to choose the most appropriate level in each dimension. Through weighing of the items, an index score between 0 and 1 was calculated, a higher score indicating a better quality of life. Only the change in scores was considered for this study.

### 2.7. Assessment of Covid-19 pandemic-associated changes of daily living

In 18 items, patients were asked about changes in their daily life due to the regulations (Appendix 2, available at http://links.lww.com/PR9/A84).

The items refer to

(1) Whether the patient lives alone and/or has pets
(2) Social interactions
(3) Medical care
(4) Activities of daily life
(5) Physical activities (usual amount of days they spent walking at least 10 minutes a day) in the past week and whether there had been changes due to the regulations.
(6) Handling of the COVID-19 news, habits of media consumption
(7) Influence of restrictive regulations on pain, sleep, mood, and fear of future
(8) Current state of employment and existential fear

For part of the analysis, patients were divided into 2 groups according to whether they experienced social change or not (see below).

### 2.8. Statistical analysis

The analysis of the collected data was performed using IBM SPSS statistics for Windows (Version 25.0). Intraindividual data of first and second assessments were compared using Wilcoxon signed rank test. Intergroup comparisons were calculated with differences in questionnaire results between first and second assessments for patients with and without social changes and with and without a pain increase using the Mann–Whitney *U*-test. “Social change” was defined in case a patient had not lived socially distanced before February and personal social contacts were discontinued due to the pandemic-associated regulations. Patients who had lived socially distanced before the pandemic already, ie, had no contact to friends and family or stated that their social life remained unchanged during the pandemic regulations, were considered for the “no social change” category. These categories were based upon questions in the pandemic questionnaire (question number 1 and 7, see Appendix 2, available at http://links.lww.com/PR9/A84).

Chi-square test was used for statistical calculation of categorical variables between first and second assessments. Values are presented as mean ± SD, and *P* < 0.05 was considered statistically significant.

## 3. Results

43 patients were included in the analysis (Fig. 1). The time of assessment was 23.1 ± SD months (range 8–36 months) after the assessment that had been performed for the former study. Patient characteristics such as sex, age, pain intensity, and comorbidities are shown in Table 1. At the time of the second assessment (=t1), 20 (46.5%) patients reported having suffered from polyneuropathy-induced pain for 1 to 5 years and 23 (53.5%) patients for more than 5 years. Most patients (n = 25, 58.1%) were diagnosed with idiopathic polyneuropathy. The polyneuropathy of 9 patients (20.9%) was caused by diabetes mellitus. 4 patients (9.3%) suffered from medication-induced polyneuropathy (2 of those caused by chemotherapy) and 2 patients (4.7%) from autoimmune-induced polyneuropathy. There was one patient each with one of the following diagnoses: polyneuropathy caused by alcohol abuse, POEMS syndrome, and vitamin B12 deficiency. None of the patients currently received chemotherapy or any other cancer treatment at either point in time. The analgesic medication is listed in Table 2, and pain locations are shown in Table 3. The number of coanalgesics did not differ significantly between t0 and t1 (t0 = 23, t1 = 30). Patients reported a general pain relief due to medication of 46.7% at t0 and 38.75% at t1 (*P* = ns). Despite the multitude of measures, we did not perceive any complaints by the patients. The feedback was overwhelmingly positive, and the patients communicated their satisfaction with the early inspection of this matter and their curiosity. 68/71 (95.8%) returned the completed questionnaires.

**Table 1:** Characteristics of patients [n = 43].

Characteristic	
Age in years [mean ± SD] (range)	66.63 ± 11.16 (43–86)
Gender [m/f] (%)	31/12 (72.1/27.9)
Average pain intensity [mean ± SD] (range) [NRS]	3.93 ± 1.88 (1–8)
Comorbidities	
Vascular (ie, stroke, heart disease, hypertension)	31 (72.1%)
Diabetes mellitus type I or II	10 (23.3%)
Cancer or precancer	7 (16.3%)
Rheumatoid	5 (11.6%)
Pulmonary (ie, COPD, asthma)	4 (9.3%)
Depression	2 (4.7%)
HIV	1 (2.3%)
Kidney disease	1 (2.3%)
Under immunosuppressive therapy	8 (18.6%)

NRS, numerical rating scale with 0 = no pain to 10 = maximal imaginable pain.

**Table 2:** Analgesic medication.

	T0	T1	p_t1 vs. t0_
Ca-antagonists (gabapentin, pregabalin)	15 (34.9%)	17 (39.5%)	ns
Na-antagonists	3 (7%)	3 (7%)	ns
Opioids	6 (14%)	8 (18.6%)	ns
NSAID*	9 (21%)	9 (21%)	ns
Tricyclic antidepressants	2 (4.7%)	3 (7%)	ns
SSNRI†	2 (4.7%)	4 (9.3%)	ns
SSRI‡	1 (2.3%)	0 (0%)	ns
Cannabinoids	0 (0%)	3 (7%)	ns
No pain medication	15 (34.9%)	13 (30.2%)	ns

* Nonsteroidal anti-inflammatory drugs.

† Serotonin–norepinephrine reuptake inhibitors.

‡ Selective serotonin reuptake inhibitors.

**Table 3:** Pain locations.

Pain location n (%)	t0	t1
Feet only	21 (48.8)	11 (25.6)
Feet and calves	7 (16.3)	19 (44.2)
Feet, calves, and thighs	13 (30.2)	12 (27.9)
Upper extremity affected	11 (25.6)	12 (27.9)
Other pain locations*	8 (18.6)	15 (34.9)
Headache	0	1 (2.2)
Neck pain	2 (4.7)	4 (9.3)
Thoracal pain	2 (4.7)	1 (2.2)
Arm/shoulder pain	2 (4.7)	6 (14.0)
(Lower) back pain	4 (9.3)	9 (20.9)
Thigh pain	0	1 (2.2)

* Likely not related to polyneuropathy.

### 3.1. Assessment of pain

#### 3.1.1. Pain intensity and characteristics

Except for the NPSI Total Score, which indicated an improvement of the extent of the neuropathic pain, the total scores and subscores of pain questionnaires did not change between the baseline assessment and the follow-up (Table 4). However, grading of BPI Scores into mild, moderate, and severe showed more patients suffering from moderate pain and less patients suffering from severe pain upon follow-up (*P* = 0.004, Table 4). In line with this finding, only 5 (11.6%) patients indicated a deterioration of their pain intensity within the first 2 weeks of the pandemic regulations (Table 5).

**Table 4:** Pain ratings and characteristics in the overall cohort.

	n*	n_t0_	n*	n_t1_	*P* [t0 vs t1]
BPI					
BPI score interpretation					0.004
Mild	42	24	43	23	
Moderate	42	11	43	17	
Severe	42	7	43	3	

	n _t0_*	t0 [mean ± SD] (range)	n _t1_*	t1 [mean ± SD] (range)	*P*
BPI					
BPI pain severity score†	42	4.04 ± 2.47 (0–10)	43	3.87 ± 1.83 (0.60–8.20)	ns
BPI worst pain in the last 24 hours	43	5.37 ± 2.55 (0–10)	43	5.28 ± 2.22 (1–9)	ns
BPI least pain in the last 24 hours	42	2.81 ± 2.64 (0–10)	43	2.49 ± 1.86 (0–7)	ns
BPI average pain in the last 24 hours	43	4.00 ± 2.56 (0–10)	43	3.93 ± 1.88 (0–8)	ns
BPI pain right now	43	3.79 ± 2.72 (0–10)	43	3.23 ± 2.19 (0–9)	ns
BPI average pain in the last 7 d	43	4.49 ± 2.44 (0–10)	43	4.44 ± 2.11 (0–9)	ns
NPSI					
NPSI total score†	37	33.54 ± 20.48 (5–93)	40	27.38 ± 16.16 (2–67)	0.008

* Number of patients who filled out the questionnaire; ns = not significant.

† Only significant items and subscores listed.

t0, first assessment; t1, 2 wk after restrictions; BPI, Brief Pain Inventory; NPSI, Neuropathic Pain Symptom Inventory.

**Table 5:** Results of pandemic-related questionnaire.

	Out of n total	n (%)
Has experienced a change in social life	43	35 (81.4)
Has experienced disadvantages in medical care since the pandemic	40	18 (45.0)
Sleep has worsened because of the pandemic	43	12 (27.9)
Appetite has worsened because of the pandemic	43	2 (4.7)
Mood has worsened because of the pandemic	43	21 (48.8)
Pain has worsened because of the pandemic	43	5 (11.6)
More worried about own health than before the pandemic	40	23 (57.5)
Worried about the course of the pandemic	40	
Little/moderately		22 (55.0)
Strongly/very strongly		11 (27.5)

#### 3.1.2. Pain interference and quality of life

Pain Catastrophizing Scale Rumination Score decreased from baseline to follow-up, ie, patients spent less time occupied with thinking about their pain.

Looking specifically at patients with stable or improved pain within the first 2 weeks of the regulations, they showed a lower PCS Rumination Score, ie, they seemed to be less occupied with their pain (Table 6). By contrast, a higher PCS Helplessness Score was found in those with a deterioration of pain (Table 6). In addition, patients with a deterioration of pain reported a lower quality of life upon EQ5D (Table 6).

**Table 6:** Comparison of patients with stable or improved pain and patients with worse pain.

	Pain stable or improved				Pain worse				Pain stable/improved vs worse (Δt0, t1)			
	n*	t0 [mean ± SD]	t1 [mean ± SD]	*P*	n*	t0 [mean ± SD]	t1 [mean ± SD]	*P*	n*	Pain stable/improved [mean ± SD]	Pain worse [mean ± SD]	*P*
PROMIS questionnaires												
PROMIS depression score	24	50.89 ± 9.36	51.40 ± 7.77	ns	17	48.61 ± 7.47	51.08 ± 7.72	ns	40	0.53 ± 6.29	1.69 ± 8.03	ns
PROMIS anxiety score	17	53.28 ± 9.79	53.32 ± 7.75	ns	10	54.05 ± 6.91	55.11 ± 7.64	ns	26	1.02 ± 7.27	1.50 ± 5.33	ns
PROMIS fatigue score	24	53.44 ± 12.27	51.58 ± 8.84	ns	18	51.33 ± 8.04	55.53 ± 9.31	ns	41	−1.38 ± 7.74	−0.80 ± 7.98	ns
PROMIS pain interference score	22	64.17 ± 8.94	63.86 ± 8.21	ns	18	58.49 ± 4.70	59.30 ± 5.76	ns	40	−1.07 ± 6.37	0.81 ± 3.85	ns
PROMIS sleep score	21	56.21 ± 8.89	56.02 ± 8.49	ns	14	51.65 ± 9.38	54.30 ± 7.18	ns	35	−0.71 ± 4.63	1.47 ± 8.53	ns
PCS												
PCS score†	25	22.24 ± 13.97	20.08 ± 11.76	ns	18	19.33 ± 9.27	21.11 ± 10.32	ns	43	−2.16 ± 10.51	1.78 ± 6.58	ns
PCS rumination score	25	8.48 ± 5.05	6.46 ± 4.27	0.011	17	6.82 ± 4.07	6.53 ± 4.68	ns	41	−2.37 ± 4.34	0.29 ± 3.84	ns
PCS helplessness score	25	9.64 ± 7.51	10.38 ± 5.11	ns	18	8 ± 4.31	10.88 ± 4.96	0.049	41	0.33 ± 6.05	2.82 ± 5.02	ns
IPIP												
IPIP emotional stability score	25	31.44 ± 8.49	31.50 ± 8.40	ns	18	34.50 ± 6.34	34.50 ± 6.63	ns	38	0.77 ± 4.13	0.38 ± 2.68	ns
EQ-5D												
EQ-5D index score	22	0.55 ± 0.21	0.57 ± 0.18	ns	18	0.66 ± 0.18	0.51 ± 0.2	0.031	40	0.03 ± 0.28	−0.15 ± 0.24	ns

* Number of patients who completed the questionnaires; ns = not significant.

† Only significant subscores listed.

t0, first assessment; t1, 2 wk after restrictions; EQ-5D, EuroQol 5 Dimensions Questionnaire; IPIP, International Personality Item Pool; PCS, Pain Catastrophizing Scale.

Neither the PROMIS Pain Interference Score nor the EQ-5D assessment (EQ-5D Index) resulted in significant changes in the overall patient cohort.

### 3.2. Assessment of emotional well-being and sleep

Interestingly, in contrast to pain intensity, a remarkable number of patients reported a worsening of their mood (21/43, 48.8%) and sleep (12/43, 27.9%) due to the pandemic in the pandemic-related questionnaire (Appendix 3, available at http://links.lww.com/PR9/A84). However, this worsening was not mirrored by reports upon self-reported, validated questionnaires (Table 7).

**Table 7:** Change of psychological parameters and pain impact on life between first assessment and follow-up.

Psychological characteristic	n_t0_*	t0 [mean ± SD] (range)	n_t1_*	t1 [mean ± SD] (range)	*P* [t0 vs t1]
PROMIS Depr.					
PROMIS depression T-score	41	49.95 ± 8.60 (38.4–70.6)	42	51.26 ± 7.66 (38.4–68.0)	ns
PROMIS depression not within normal limits n (%)	41	11 (27.5%)	42	15 (37.5%)	ns
PROMIS anxiety					
PROMIS anxiety T-score	27	53.57 ± 8.70 (39.1–72.1)	41	54.11 ± 7.66 (39.1–71.3)	ns
PROMIS anxiety not within normal limits n (%)	27	11 (40.7%)	41	11 (26.8%)	ns
PROMIS sleep					
PROMIS sleep T-score	34	54.21 ± 9.28	34	55.35 ± 7.97	ns
PROMIS sleep not within normal limits n (%)	34	18 (52.9%)	34	15(44.1%)	ns
PROMIS fatigue					
PROMIS fatigue T-score	42	52.54 ± 10.60 (33.7–75.8)	41	51.12 ± 8.95 (33.7–69.0)	ns
PROMIS fatigue not within normal limits n (%)	42	17 (40.5%)	41	15 (36.6%)	ns
PROMIS pain interference					
PROMIS pain interference T-score	42	61.67 ± 7.84	42	61.91 ± 7.53	ns
PCS					
PCS rumination	42	7.81 ± 4.70 (0–16)	41	6.49 ± 4.39 (0–14)	**0.030**
PCS magnification	41	4.66 ± 2.43 (0–9)	42	4.33 ± 2.75 (0–10)	ns
PCS helplessness	43	8.95 ± 6.35 (0–24)	41	10.59 ± 5.00 (2–21)	ns
PCS total score	43	21.10 ± 12.27 (0–48)	43	21.05 ± 10.93 (4–45)	ns
IPIP					
IPIP emotional stability	43	32.72 ± 7.73 (15–45)	38	32.76 ± 7.75 (14–43)	ns
EQ-5D					
EQ-5D index score	42	0.60 ± 0.20	41	0.55 ± 0.19	ns

* Number of patients who completed the questionnaire.

t0, first assessment; t1, 2 wk after restrictions; IPIP, International Personality Item Pool; ns, not significant; PCS, Pain Catastrophizing Scale.

### 3.3. Assessment of Covid19-associated changes of daily living

A set of pandemic-associated changes are shown in Table 5. The results of the remaining items of the COVID-associated questionnaire, that have been collected to characterize the study cohort, are shown in Appendix 3 (available at http://links.lww.com/PR9/A84). There was no significant change measurable concerning the number of days the patients spent walking more than 10 minutes in the past week (t0: 5.10 ± 2.40; t1: 4.90 ± 2.52; *P* = ns). Out of 40, 33 (82.5%) still reported to go out for walks and 35/40 (87.5%) reported to do some exercise at home. More than 50% of the patients used to exercise regularly outside home before the pandemic, which was not possible anymore due to the regulations (Appendix 3, available at http://links.lww.com/PR9/A84).

18 patients (45.0%) reported having experienced disadvantages in medical care due to the pandemic. Those disadvantages affected doctors' appointments (n = 13, 32.5% of all patients who have answered the question), drug supply (n = 7, 17.5% of all patients who have answered the question), and postponed surgical interventions (n = 1, 2.5%, details in Appendix 3, available at http://links.lww.com/PR9/A84).

Patients who did not report any medical disadvantages due to the pandemic showed a lower NPSI Total Score (t0: 31.21 ± 21.46 vs t1: 22.75 mean ±14.5 SD; *P* = 0.041). They also showed an increase in the IPIP-Emotional Stability Score (t0: 33.27 ± 7.6 vs t1: 35.00 ± 6.97; *P* = 0.021) and an increase in the PROMIS Anxiety Score (t0: 51.22 ± 8.11 vs t1: 52.59 ± 5.6; *P* = 0.023).

### 3.4. Influence of social isolation

**Figure 2.**
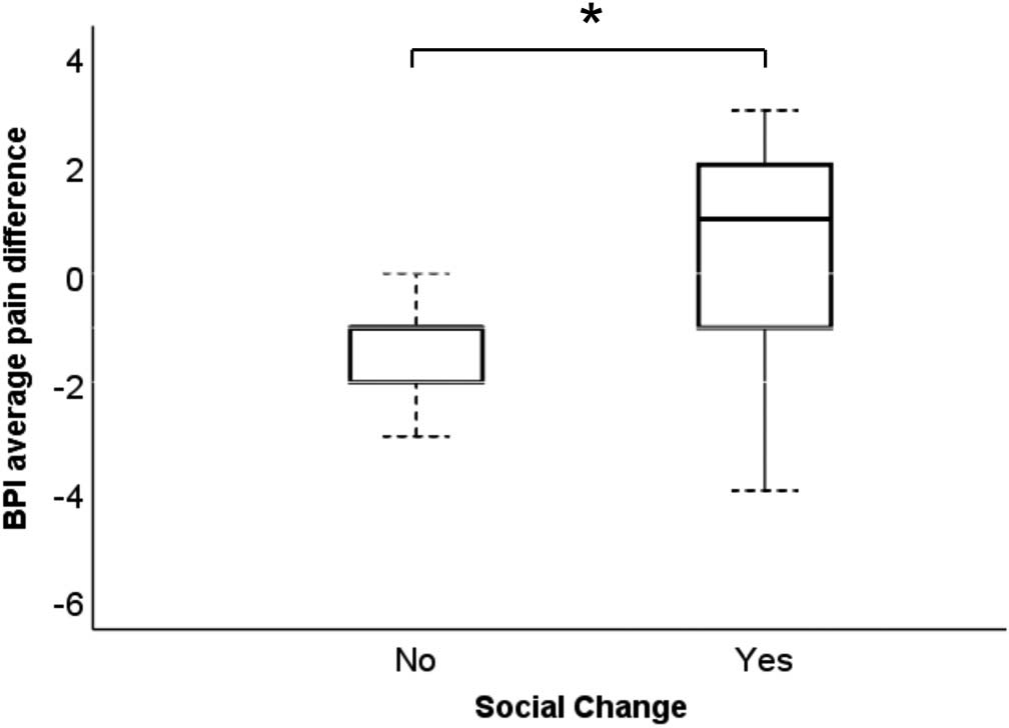
*Difference of the average pain during the past 7 days according to the Brief Pain Inventory (BPI) between baseline and follow-up in the patient group with and without social change.

Through the COVID-related items, 35 patients (81.4%) described a changed social life during the pandemic. Patients with a steady social life presented an improvement or a trend towards an improvement of pain intensity upon several pain scales in the first 2 weeks of the pandemic regulations (Table 8), whereas patients with a social change showed higher pain ratings compared to those without a social change on BPI average pain rating within the last 7 days (Fig. 2 and Table 8).

**Table 8:** Display of different parameters in the social change-dependent subgroups [n = 43].

	No social change			Social change			No social change vs social change (Δt0, t1)		
	n*	Mean ± SD	*P*	n*	Mean ± SD	*P*	n*	Mean ± SD	*P*
BPI									
BPI worst pain in the last 24 hours	8	t0: 4.75 ± 2.05 t1: 3.13 ± 1.81	0.024	35	t0: 5.51 ± 2.65 t1: 5.77 ± 2.02	ns	8	−1.63 ± 2.00 vs 0.26 ± 2.57	ns
BPI least pain in the last 24 hours	7	t0: 1.57 ± 1.40 t1: 1.00 ± 1.31	ns	35	t0: 3.06 ± 2.78 t1: 2.83 ± 1.81	ns	7	−0.043 ± 0.98 vs −0.23 ± 1.72	ns
BPI average pain in the last 24 hours	8	t0: 3.50 ± 2.27 t1: 2.13 ± 1.46	0.011	35	t0: 4.11 ± 2.64 t1: 4.34 ± 1.73	ns	8	−1.38 ± 1.51 vs 0.23 ± 1.86	0.033
BPI pain right now	8	t0: 2.75 ± 1.75 t1: 0.25 ± 0.46	0.018	35	t0: 4.03 ± 2.86 t1: 3.91 ± 1.82	ns	8	−2.50 ± 1.78 vs −0.11 ± 1.89	0.004
BPI average pain in the last 7 d	8	t0: 3.88 ± 2.23 t1: 2.25 ± 1.75	0.014	35	t0: 4.63 ± 2.50 t1: 4.94 ± 1.86	ns	8	−1.63 ± 1.60 vs 0.31 ± 1.83	0.010
BPI pain severity score†	7	t0: 3.32 ± 1.65 t1: 1.75 ± 1.05	0.018	35	t0: 4.18 ± 2.60 t1: 4.36 ± 1.61	ns	7	−1.44 ± 0.98 vs 0.18 ± 1.74	0.038
NPSI									
NPSI total	7	t0: 24.86 ± 18.78 t1: 15.71 ± 11.34	ns	30	t0: 35.57 ± 20.62 t1: 29.85 ± 16.07	0.028	7	−9.14 ± 12.25vs −6.68 ± 14.27	ns
PCS									
PCS score†	8	t0: 18.25 ± 14.42 t1: 12.63 ± 8.54	ns	35	t0:21.66 ± 11.76 t1: 22.31 ± 10.87	ns	8	−5.62 ± 9.72 vs 0.66 ± 8.80	ns
PCS rumination score	7	t0: 7.5 ± 6.05 t1: 4.43 ± 2.99	0.046	34	t0: 7.88 ± 4.43 t1: 6.91 ± 4.54	ns	7	−4.14 ± 3.58 vs −0.68 ± 4.24	0.023
PCS helplessness score	7	t0: 6.88 ± 5.79 t1: 7.00 ± 3.23	ns	35	t0: 9.43 ± 6.46 t1: 11.32 ± 5.00	ns	7	−0.86 ± 5.98 vs 1.82 ± 5.64	ns
EQ-5D									
EQ-5D index score	6	t0: 0.76 ± 0.05 t1: 0.55 ± 0.2	0.043	35	t0: 0.57 ± 0.20 t1: 0.55 ± 0.19	ns	5	−0.25 ± 0.19 vs −0.02 ± 0.28	ns

* Number of patients who completed the questionnaires.

† Only significant single items and subscores listed.

BPI, Brief Pain Inventory; EQ-5D, EuroQol 5 Dimensions Questionnaire; NPSI, Neuropathic Pain Symptom Inventory; ns, not significant; PCS, Pain Catastrophizing Scale; t0, first assessment; t1, 2 wk after restrictions.

Patient Reported Outcomes Measurement Information System depression, anxiety, pain interference, sleep, and fatigue scores, and quality of life did not differ between those with and without a change of social environment. The PCS Rumination Score was higher in patients with a change of social environment compared to those without (Table 8).

## 4. Discussion

Two weeks after the onset of the pandemic-associated regulations, only 11.6% of patients reported a worsening of pain intensity, whereas 82.5% stated to be worried about the course of the pandemic. Accordingly, mean pain intensity remained stable or even improved, whereas PCS Rumination Score decreased. Patients who experienced a change of social life consequently to the regulations had increased pain ratings, reported less quality of life, and demonstrated more pain catastrophizing thoughts. Overall, however, the effect of the pandemic regulations on pain intensity was—contrary to the expected—at best mild, although the regulations significantly impacted daily life. Given that the patients would undergo the often-reported, common patterns of disaster response,^12^ a possible explanation is our early assessment. The present results would then mirror the so-called “heroic” stage which would be characterised through provisional adjustment. Accordingly, a disillusionment on the further time course would be expected, characterised by resentment and uncertainty. We therefore hypothesize that collecting data at forthcoming dates is likely to reveal more distinct changes.

Eccleston et al. state that the pandemic will have consequences for patients with chronic pain conditions, discussing that this might also be due to diversion of resources and the circumstance that many healthcare professionals specialising in pain are directly relevant for the acute response to the pandemic.^14^ Accordingly, nearly half of our study cohort reported to have experienced medical disadvantages since the pandemic. Those who did not, however, displayed a decrease in neuropathic pain and a higher IPIP emotional stability score (probably feeling confident as they did not experience the medical disadvantages that had been predicted to occur).

The overall decrease of the NPSI Total Score and PCS Rumination Score suggests that patients spent less time thinking about their pain, in line with the BPI Scores that showed more patients suffering from moderate pain and less patients suffering from severe pain. We presume this might be due to the circumstance that the emergence of a pandemic (associated with anxiety, overflowing information, and dramatic changes in public and private life) poses a “distraction” from the chronic pain condition.^2,32,34^ Comparing the results for patients with and without a worsening of pain, those without had lower scores upon several PCS items (eg, rumination). Patients with a worsening of pain showed higher PCS helplessness scores and indicated to have a lower quality of life—implying that a certain predisposition to catastrophize might be linked to higher pain perception. A connection between increased behavioural expressions of pain and catastrophizing^36^ and a contribution of a tendency to “catastrophize” during painful stimulation to more intense pain experience have been described earlier.^39^ Thus, coping mechanisms might be distinctive tools for the management of pain during the pandemic.

In addition, patients with a social change reported higher or stable pain ratings, whereas patients without a social change even presented a trend towards an improvement of the pain. Results of stable or even improved pain intensity can again be explained by distraction from pain, whereas increased pain intensity in those with social change is in line with findings by Karayannis et al.^26^ who showed that the impact of pain is reduced in individuals who have the feeling of being included and engaged with others. A link of chronic and temporary loneliness with increased physical pain had been shown for fibromyalgia patients.^46^ The lower PCS Rumination Scores in patients with a steady social life stress the importance of maintaining contact with the family and social entourage in times of pandemic-associated regulations.^5,22^ Although the majority of our cohort reportedly remained in contact through telephone, personal interaction clearly is irreplaceable. Thus, social integration also seems to be important for the handling of pain during the pandemic.

Wide-ranging psychological consequences of comparable events leading to quarantine and isolation (eg, anxiety through financial loss,^5,25^ depressive disorders^30^) have been reported. During the initial phase of the SARS-CoV-2 outbreak in China, the psychological impact was rated as moderate to severe by more than half of the respondents,^43^ and a high prevalence of low mood (73.1%) has been described.^29^ Although nearly half of our patients indeed reported a worsened mood due to the regulations in our specific COVID-19-related items, we did not observe an influence on psychological parameters upon self-reported questionnaires, possibly due to above-mentioned short time interval between regulations and assessment and because these questionnaires have not been validated specifically under pandemic regulations.

Quality of life 2 weeks after the onset of the regulations has not been affected significantly in our overall study population. This again is explainable through the assessment time. The examined time frame was accompanied by an immense public esprit of cohesion and “being in this together,” possibly contributing to a certain appeasement of worries and fears. In addition, more than half of the study population (64.1%) indicated to be confident that the situation would improve in the coming weeks and only 2 patients reported existential fears. It will be of great interest to examine pain intensity, emotional well-being, and quality of life amid the upcoming fake news, conspiracy theories, and economic pressure.

This work draws its relevance from the fact that chronic pain is a prominent health issue that reportedly affects up to one-fifth of the world's population^23^ with a bidirectional influence of similar magnitude of pain and mental illness.^4^ Bearing in mind that depression has been reported to be one of the leading contributors to the global disease burden^11,44^ and contributes to pain deterioration, patients with a chronic pain condition clearly require special attention amid the expected psychological impact of the pandemic.

One limitation of this study is that the first assessment was part of former studies and thus not directly before the pandemic regulations. Thus, we cannot fully conclude that the observed changes are really a consequence of the pandemic regulations or due to a memory bias of patients. However, to minimize this bias, we have included only patients who reported a stable disease between first and second assessments. In addition, treatment medication might have influenced the outcome. We acknowledge that the comparison of patients with and without social change is underpowered (with only 8 patients without social change), but nonetheless found the comparison to be of interest. However, it is important to recognize that this study is exploratory due to the unique situation and needs confirmation in targeted studies.

## 5. Conclusion

In the current assessment, 11.6% indicated that their pain worsened due to the pandemic, as opposed to 82.5% that stated to be worried about the course of the pandemic. A significant decrease of ruminating on pain and of the NPSI Score in painful neuropathy patients was observed 2 weeks after the onset of regulations, suggesting that pain stands back amid the very real threat of a devastating pandemic. With the whole public and private life orientated towards tackling the exceptional situation and the media continuously reporting about various aspects of it, the population's attention is focused on the pandemic and is most likely to be in the “heroic” phase. Consequently, it remains to be of interest how pain and mental health of the patients will evolve in the forthcoming weeks with persisting, exceptional impacts on public and private life.

## Disclosures

This study was supported by Grünenthal GmbH Deutschland with no involvement in experimental design, conduction, or interpretation of the study. Dilara Kersebaum reports grants from Grünenthal GmbH, during the conduct of the study; Sophie-Charlotte Fabig reports grants from Grünenthal GmbH, during the conduct of the study as well as personal fees from Grünenthal GmbH outside the submitted work; Manon Sendel reports grants from Grünenthal GmbH, during the conduct of the study; personal fees from Sanofi Genzyme, personal fees from Grünethal, personal fees from AKCEA, outside the submitted work; Dr. Sachau reports grants from Grünenthal GmbH, during the conduct of the study; non-financial support from Alnylam Pharmaceuticals, non-financial support from Pfizer, personal fees from Grünenthal GmbH, outside the submitted work;. Josephine Lassen reports grants from Grünenthal GmbH, during the conduct of the study; Dr. Rehm reports grants from Grünenthal GmbH, during the conduct of the study; Dr. Huellemann reports grants from Grünenthal GmbH, during the conduct of the study; grants from BMBF, grants from Medoc , grants from Zambon, outside the submitted work; Dr. Baron reports grants from Grünenthal GmbH, during the conduct of the study; grants from EU Projects: “Europain” (115007). DOLORisk (633491). IMI Paincare (777500). German Federal Ministry of Education and Research (BMBF): Verbundprojekt: Frühdetektion von Schmerzchronifizierung (NoChro) (13GW0338C). German Research Network on Neuropathic Pain (01EM0903). Pfizer Pharma GmbH, Genzyme GmbH, Grünenthal GmbH, Mundipharma Research GmbH und Co. KG., Novartis Pharma GmbH, Alnylam Pharmaceuticals Inc., Zambon GmbH, personal fees from Pfizer Pharma GmbH, Genzyme GmbH, Grünenthal GmbH, Mundipharma, Sanofi Pasteur, Medtronic Inc. Neuromodulation, Eisai Co.Ltd., Lilly GmbH, Boehringer Ingelheim Pharma GmbH & Co. KG, Astellas Pharma GmbH, Desitin Arzneimittel GmbH, Teva GmbH, Bayer-Schering, MSD GmbH, Seqirus Australia Pty. Ltd, Novartis Pharma GmbH, TAD Pharma GmbH, Grünenthal SA Portugal, Sanofi-Aventis Deutschland GmbH, Agentur Brigitte Süss, Grünenthal Pharma AG Schweiz, Grünenthal B.V. Niederlande, personal fees from Pfizer Pharma GmbH, Genzyme GmbH, Grünenthal GmbH, Mundipharma Research GmbH und Co. KG, Allergan, Sanofi Pasteur, Medtronic, Eisai, Lilly GmbH, Boehringer Ingelheim Pharma GmbH&Co.KG, Astellas Pharma GmbH, Novartis Pharma GmbH, Bristol-Myers Squibb, Biogenidec, AstraZeneca GmbH, Merck, Abbvie, Daiichi Sankyo, Glenmark Pharmaceuticals S.A., Seqirus Australia Pty. Ltd, Teva Pharmaceuticals Europe Niederlande, Teva GmbH, Genentech, Mundipharma International Ltd. UK, Astellas Pharma Ltd. UK, Galapagos NV, Kyowa Kirin GmbH, Vertex Pharmaceuticals Inc., Biotest AG, Celgene GmbH, Desitin Arzneimittel GmbH, Regeneron Pharmaceuticals Inc. USA, Theranexus DSV CEA Frankreich, Abbott Products Operations AG Schweiz, Bayer AG, Grünenthal Pharma AG Schweiz, Mundipharma Research Ltd. UK, Akcea Therapeutics Germany GmbH, Asahi Kasei Pharma Corporation, AbbVie Deutschland GmbH & Co. KG, Air Liquide Sante International Frankreich, Alnylam Germany GmbH, Lateral Pharma Pty Ltd, Hexal AG, Ethos Srl Italien, Janssen, outside the submitted work; Dr. Gierthmühlen reports grants from Grünenthal GmbH, during the conduct of the study; personal fees from TAD Pharma, personal fees from Glenmark, Certkom, personal fees from Pfizer, Grünenthal, Sanofi Pasteur, Novartis, outside the submitted work.

## Appendix A. Supplemental digital content

Supplemental digital content associated with this article can be found online at http://links.lww.com/PR9/A84.
